# Rapid prediction of carbapenemases in *Pseudomonas aeruginosa* by imipenem/relebactam and MALDI-TOF MS

**DOI:** 10.1128/jcm.01105-24

**Published:** 2025-03-25

**Authors:** Ana Candela, María Fernández-Billón, Pablo Aja-Macaya, Lucía González-Pinto, Pablo Arturo Fraile-Ribot, Esther Viedma, Isaac Alonso-García, Tania Blanco-Martín, Roberto Estévez-Alfaya, Ana Fernández-González, Alejandro Beceiro, Carla López-Causapé, Marina Oviaño, Germán Bou, Antonio Oliver, Jorge Arca-Suárez

**Affiliations:** 1 Servicio de Microbiología and Instituto de Investigación Biomédica A Coruña (INIBIC), Complexo Hospitalario Universitario A Coruña 16811 , A Coruña, Spain; 2 Servicio de Microbiología and Unidad de Investigación, Hospital Universitario Son Espases and Instituto de Investigación Sanitaria Illes Balears 375118, Palma de Mallorca, Spain; 3 CIBER de Enfermedades Infecciosas (CIBERINFEC), Instituto de Salud Carlos III 38176 , Madrid, Spain; 4 Universidade da Coruña, A Coruña, España; 5 Servicio de Microbiología, Hospital Universitario 12 de Octubre 16473 , Madrid, Spain; Endeavor Health, Evanston, Illinois, USA

**Keywords:** MALDI-TOF, antimicrobial resistance, imipenem/relebactam, *Pseudomonas aeruginosa*, clinical microbiology

## Abstract

*Pseudomonas aeruginosa* is a major nosocomial pathogen commonly involved in multidrug-resistant (MDR) infections that are very difficult to treat. Imipenem/relebactam is a new carbapenem/β-lactamase inhibitor combination with robust activity against *P. aeruginosa*. However, resistance is increasingly reported, and rapid detection is, therefore, crucial so that appropriate treatments can be prescribed. We have developed a rapid MALDI TOF-MS-based method that can accurately predict the presence of carbapenemases in *P. aeruginosa* using imipenem/relebactam. The method was developed using a retrospective and a prospective collection of 419 *P*. *aeruginosa* isolates (including recombinant isolates and WGS-characterized clinical strains) encompassing the most important β-lactam resistance mechanisms. The MALDI TOF-MS method is based on the detection of the hydrolysis of imipenem in the presence or absence of relebactam, measuring modifications in the mass spectra of imipenem after incubation with bacteria. The method was evaluated against a retrospective collection and then validated against 250 prospectively collected clinical isolates, showing a 98% (246/250) agreement between the phenotype and the MALDI-TOF MS hydrolysis result and a 100% accordance with the β-lactam resistance genotype. Some errors in detecting GES-producing isolates and in detecting different mutational resistance mechanisms associated with imipenem/relebactam resistance (MICs ranging from 4 to 8 mg/L) were observed. All results were obtained within 1 hour, positioning the MALDI-TOF-based test as a rapid and easy-to-perform method for detection of carbapenemases (except GES enzymes) in *P. aeruginosa*. Besides, implementation of the method in routine laboratory screening would facilitate the correct use of imipenem/relebactam to treat *P. aeruginosa* infections.

**IMPORTANCE:**

While several rapid diagnostic methods have been developed for the detection of ESBLs and carbapenemases to improve treatment decision-making in Enterobacterales, there is a lack of approaches to rapidly identify resistance mechanisms and predict β-lactam susceptibility in *Pseudomonas aeruginosa*. Taking advantage of the mechanism of action and the high efficacy of the newly developed β-lactam/β-lactamase inhibitor combination imipenem/relebactam against *P. aeruginosa*, we developed a WGS-guided, MALDI-TOF-based algorithm that accurately predicts the presence of carbapenemase enzymes in this bacterium and aids in forecasting the imipenem/relebactam susceptibility profile. The implementation of this method in routine laboratory testing would provide significant support in the rapid decision-making for the use of imipenem/relebactam in severe *P. aeruginosa* infections.

## INTRODUCTION

*Pseudomonas aeruginosa* is a major nosocomial pathogen commonly involved in multidrug-resistant (MDR) and/or extensively drug-resistant (XDR) infections in immunocompromised hosts or critically ill patients and is associated with significant morbidity and mortality. This therapeutic challenge results from the overwhelming ability of *P. aeruginosa* to limit the effectiveness of all conventional β-lactam antibiotics via the selection of chromosomally encoded resistance mechanisms (e.g., upregulation of *ampC* and efflux pumps in combination with the inactivation of the outer membrane porin OprD) or via horizontal acquisition of broad-spectrum resistance elements, such as extended-spectrum beta-lactamases (ESBLs) and carbapenemases. This growing concern is further aggravated by the scarcity of newly available antibiotics with potent antipseudomonal activity and the progressive emergence in hospitals worldwide of international epidemic MDR/XDR *P. aeruginosa* clones (also known as high-risk clones) (1). This situation has been partly mitigated in recent years by the introduction of innovative cephalosporin/β-lactamase inhibitor combinations, ceftolozane/tazobactam and ceftazidime/avibactam in the clinical setting, which are stable against the main mutational mechanisms and ESBLs found in *P. aeruginosa*. However, since these agents have become available for clinical use, resistance has increasingly been described (2–4). Thus, new antimicrobial agents are urgently needed to complete the antipseudomonal armamentarium.

Imipenem/relebactam is a new carbapenem/β-lactamase inhibitor with demonstrated efficacy against MDR Gram-negative infections, including *P. aeruginosa*, as observed in two randomized clinical trials (RESTORE-IMI 1 and RESTORE-IMI 2) (5, 6). The robust activity of this new combination against *P. aeruginosa* relies on the potent antipseudomonal activity of imipenem (not lysed by the intrinsic AmpC and poorly recognized by most of *P. aeruginosa* RND-type efflux systems) in combination with relebactam, a novel diazabicyclooctane (DBO)-type β-lactamase inhibitor. Relebactam inactivates class A and C β-lactamases and is equipped with a highly reactive piperidine ring at the 2-position carbonyl group that apparently confers enhanced resistance to drug extrusion (7, 8). The addition of relebactam reduces modal imipenem minimum inhibitory concentrations (MICs) by fourfold and eightfold against imipenem-susceptible and non-susceptible *P. aeruginosa* clinical isolates, respectively (9). On the other hand, metallo-β-lactamases or class D carbapenemases, which are increasingly linked to *P. aeruginosa* epidemic strains worldwide, are not inhibited by relebactam (10).

Rapid matrix-assisted laser desorption/ionization time-of-flight mass spectrometry (MALDI-TOF)-based laboratory methods aimed at predicting susceptibility or resistance or detecting broad-spectrum β-lactam resistance mechanisms (such as ESBLs and carbapenemases) have been demonstrated to have a major impact on precise treatment decision-making, successful infection management, and patient outcomes (11). In this regard, our group and other researchers have previously developed different MALDI-TOF-based methodologies able to rapidly identify the production of ESBLs and carbapenemases in Enterobacterales (12, 13). However, given the high complexity of *P. aeruginosa* β-lactam resistance, including the presence of many β-lactamase-independent β-lactam resistance mechanisms (e.g. efflux upregulation, porin deficiency, and PBP modification), methods that rapidly predict susceptibility or infer underlying mechanisms of resistance have not yet been developed. Thus, taking advantage of the mechanism of action of imipenem/relebactam against *P. aeruginosa*, we developed an innovative MALDI-TOF-based assay to rapidly predict the presence of carbapenemase enzymes and address the gap in the availability of rapid diagnostic methods to combat MDR/XDR *P. aeruginosa* infections.

## MATERIALS AND METHODS

### Conceptualization of the algorithm for prediction of carbapenemase enzymes

The algorithm was conceptually designed on the basis of the particular mechanism of action of imipenem/relebactam against *P. aeruginosa* and considers the following: i) the well-known hydrolytic profile of imipenem, in which hydrolysis by class A, B, and D carbapenemases can be precisely monitored using MALDI-TOF (14); ii) the ability of relebactam to effectively block all AmpC molecules available in the periplasmic space and recover the susceptibility to imipenem against *P. aeruginosa,* even in the absence of a functional OprD outer membrane porin (the most common pathway for the internalization of carbapenems in *P. aeruginosa*), as well as the minimal effect of efflux on imipenem, suggesting that the activity of the main RND-type efflux pumps in *P. aeruginosa* does not significantly increase the MIC of imipenem (15); iii) the good performance of relebactam in protecting imipenem from hydrolysis by class A carbapenemases (and its inefficacy in protecting against class B or D carbapenemase variants) (16). Thus, the analysis in parallel of the hydrolysis spectra of imipenem and imipenem/relebactam would allow prediction of the presence of a carbapenemase enzyme and provide useful information about the underlying mechanisms of resistance to β-lactams. Of note, the prediction algorithm is built upon European Committee on Antimicrobial Susceptibility Testing (EUCAST) guidelines as the primary comparator for susceptibility breakpoints. A comprehensive representation of this interpretation algorithm is detailed in Table 1.

**TABLE 1:** Correlations between the genotype, phenotype, and MALDI-TOF MS susceptibility testing of imipenem and imipenem/relebactam in *P. aeruginosa*

Expected phenotype	MIC imipenem (R > 4 mg/L)^*a*^	MIC imipenem/relebactam (R > 2/4 mg/L)^*a*^	Imipenem hydrolysis^*b*^	Imipenem/relebactam hydrolysis^*b*^
Wild-type	S	S	–	–
Mutational resistance (OprD-)	R	S/R	–	–
Class A carbapenemase (KPC)	R	S	+	–
Class A carbapenemase (GES)	R	R	+/–^*c*^	+/–
Class B carbapenemase (MBL)	R	R	+	+
Class D carbapenemase (OXA-48)	R	R	+	+

^*a*^ Expected susceptibility profile (resistant, R and susceptible, S) for each phenotype when applying imipenem and imipenem/relebactam breakpoints following EUCAST guidelines v14.0.

^*b*^ Expected MALDI-TOF MS imipenem hydrolysis result (positive +, negative –) for each phenotype.

^*c*^ "+/-": intermediate hydrolysis.

### Bacterial isolates

#### Training set

To analyze the robustness of our predictive algorithm, we took advantage of the availability of three different and representative collections in our laboratories, which included up to 169 *P*. *aeruginosa* isolates: i) A collection including 17 isogenic *P. aeruginosa* PAO1-derived mutants obtained in previous work via selection in antibiotic-containing plates (spontaneous mutant) or the *cre-lox* system for gene deletion (knockout mutant) and covered the most relevant mutational β-lactam resistance mechanisms, also including combinations (Table 2) (17–19); ii) A collection of 31 PAO1-derived isolates expressing in the pUCP24 plasmid the most relevant β-lactamase genes circulating in *P. aeruginosa* (Table 3) (20–22). iii) A challenge set of 121 genetically diverse MDR/XDR WGS-characterized clinical *P. aeruginosa* contemporary isolates collected by the Clinical Microbiology Departments of two large (>1,000 bed) tertiary care teaching hospitals in Spain: the A Coruña University Hospital Complex (CHUAC) (A Coruña, Galicia) and the Son Espases University Hospital (HUSE) (Palma de Mallorca, Mallorca) (Table 4). The latter strains covered a representative array of the β-lactam resistance mechanisms most commonly found in *P. aeruginosa,* including classic mutations in chromosomal genes or production of ESBLs and carbapenemases. Complete genomic information is detailed in Table S1.

**TABLE 2:** Description of the 17 *P. aeruginosa* PAO1-derived isogenic mutants used in the training set

Isolate ID	Resistance mechanism ^*a*^	IMI (mg/L)^*b*^	RH IMI ^*c*^	IMR (mg/L) ^*b*^	RH IMR^*c*^	IMR EUCAST (R > 2 mg/L) ^*d*^	IMR MALDI ^*e*^
PAO1	Wild-type	0.5	0.02	0.125	−0.16	S	Negative
PAO Δ*dacB*	PAO1 *dacB* (PBP4) knockout mutant [↑*ampC* ≈ 50-fold]	1	−0.03	0.125	0.02	S	Negative
PAO *ΔdacBΔdacC*	PAO1 dual *dacB-dacC* (PBP4-PBP5) knockout mutant [↑*ampC* ≈ 500-fold]	1	−0.32	0.125	−0.19	S	Negative
PAO *ΔdacBΔdacCΔpbpG*	PAO1 triple *dacB-dacC-dacG* (PBP4-PBP5-PBP7) knockout mutant [↑*ampC* ≈ 1,200-fold]	0.125	−0.21	0.125	−0.27	S	Negative
PAO *ΔampD*	PAO1 *ampD* knockout mutant [↑*ampC* ≈ 50-fold]	0.5	0.08	0.125	−0.16	S	Negative
PAO *ΔampDΔampDh2ΔampDh3*	PAO1 *ampD-ampDh2-ampDh3* knockout mutant [↑*ampC* ≈ 1,000-fold]	0.5	−0.13	0.0625	−0.13	S	Negative
PAO *ΔdacBΔampD*	PAO1 *dacB* (PBP4)-*ampD* knockout mutant [↑*ampC* ≈ 1,500-fold]	1	−0.15	0.125	−0.27	S	Negative
PAO Δ*oprD*	PAO1 spontaneous *oprD-*deficient mutant	8	0.04	0.25	−0.03	S	Negative
PAO *ΔampCΔoprD*	PAO1 *ampC* knockout *oprD*-deficient mutant	2	−0.06	0.5	−0.08	S	Negative
PAO *ΔmexR*	PAO1 *mexR* knockout mutant [↑*mexB* ≈ 10-fold]	0.5	−0.30	0.125	−0.17	S	Negative
PAO *ΔnfxB*	PAO1 *nfxB* knockout mutant [↑*mexD* ≈ 150-fold]	0.5	−0.07	0.125	−0.04	S	Negative
PAO *ΔmexZ*	PAO1 *mexZ* knockout mutant [↑*mexY* ≈ 15-fold]	1	−0.04	0.125	−0.20	S	Negative
PAO *ΔampDΔmexR*	PAO1 *ampD-mexR* knockout mutant [↑*ampC* ≈ 50-fold + *mexB* ≈ 10-fold]	0.5	0.17	0.125	−0.27	S	Negative
PAO *ΔampDΔoprD*	PAO1 *ampD* knockout *oprD*-deficient mutant [↑*ampC* ≈ 50-fold + OprD–]	16	−0.06	0.5	−0.23	S	Negative
PAO *ΔdacBΔoprD*	PAO1 *dacB* knockout *oprD*-deficient mutant [↑*ampC* ≈ 50-fold + OprD–]	16	−0.49	0.5	−0.11	S	Negative
PAO *ΔmexRΔoprD*	PAO1 *mexR* knockout *oprD*-deficient mutant [↑*mexB* ≈ 10-fold + OprD–]	8	−0.09	0.5	0.00	S	Negative
PAO *ΔmexZΔoprD*	PAO1 *mexZ* knockout *oprD*-deficient mutant [↑*mexY* ≈ 15-fold + OprD–]	16	0.14	1	−0.05	S	Negative

^*a*^ Description of mutational β-lactam resistance mechanisms in isogenic *P. aeruginosa* PAO1-derived mutants.

^*b*^ MIC (mg/L) of imipenem (IMI) and imipenem/relebactam (IMR).

^*c*^ Ratio of hydrolysis (RH) of imipenem (IMI) and imipenem/relebactam (IMR) obtained by MALDI-TOF MS.

^*d*^ Interpretation of susceptibility/resistance of imipenem/relebactam (IMR) according to EUCAST guidelines.

^*e*^ Interpretation of the hydrolysis of imipenem/relebactam (IMR) by MALDI-TOF MS.

**TABLE 3:** Description of 31 PAO1-derived *P. aeruginosa* isolates expressing in the pUCP24 plasmid the most relevant β-lactamase genes circulating in *P. aeruginosa*

Isolate ID^*a*^	Phenotype^*b*^	Ambler class	IMI (mg/L)^*c*^	RH IMI^*d*^	IMR (mg/L) ^*c*^	RH IMR ^*d*^	IMR EUCAST (R > 2 mg/L) ^*e*^	IMR_MALDI ^*f*^
PAO1 GES-1	ESBL	A	1	−0.10	0.5	−0.20	S	Negative
PAO1 GES-5	Carbapenemase	A	2	−0.82	0.5	−0.02	S	Negative
PAO1 GES-15	ESBL	A	1	−0.30	0.5	−0.10	S	Negative
PAO1 GES-7	ESBL	A	1	0.10	0.25	−0.50	S	Negative
PAO1 GES-20	Carbapenemase	A	1	−0.95	0.5	−0.13	S	Negative
PAO1 PER-1	ESBL	A	1	−0.31	0.5	−0.26	S	Negative
PAO1 VEB-1	ESBL	A	1	−0.08	0.5	−0.13	S	Negative
PAO1 KPC-2	Carbapenemase	A	64	1.03	1	−0.10	S	Negative
PAO1 KPC-3	Carbapenemase	A	64	1.04	1	−0.08	S	Negative
PAO1 KPC-35	ESBL	A	1	−0.24	0.25	−0.14	S	Negative
PAO1 KPC-31	ESBL	A	1	−0.15	0.5	−0.14	S	Negative
PAO1 CTX-M-15	ESBL	A	1	−0.22	0.25	0.07	S	Negative
PAO1 CTX-M-9	ESBL	A	1	−0.44	0.25	−0.23	S	Negative
PAO1 SHV-12	ESBL	A	1	−1.16	0.25	−0.20	S	Negative
PAO1 FOX-4	Extended-spectrum cephamycinase	C	1	0.04	0.5	−0.18	S	Negative
PAO1 CMY-2	Extended-spectrum cephamycinase	C	2	−0.95	0.25	−0.14	S	Negative
PAO1 DHA-1	Extended-spectrum cephamycinase	C	1	−0.09	0.5	−0.05	S	Negative
PAO1 IMP-13	Carbapenemase	B	8	0.78	8	0.57	R	Positive
PAO1 IMP-94	Carbapenemase	B	8	1.50	8	1.18	R	Positive
PAO1 NDM-1	Carbapenemase	B	32	1.17	32	0.93	R	Positive
PAO1 NDM-5	Carbapenemase	B	128	1.10	128	1.07	R	Positive
PAO1 NDM-7	Carbapenemase	B	>128	0.95	>128	1.16	R	Positive
PAO1 NDM-23	Carbapenemase	B	32	0.86	32	1.22	R	Positive
PAO1 VIM-1	Carbapenemase	B	16	0.51	8	0.62	R	Positive
PAO1 VIM-2	Carbapenemase	B	8	1.05	8	1.01	R	Positive
PAO1 VIM-20	Carbapenemase	B	32	1.00	16	1.11	R	Positive
PAO1 OXA-2	Narrow-spectrum oxacillinase	D	4	−1.22	1	−0.09	S	Negative
PAO1 OXA-10	Narrow-spectrum oxacillinase	D	2	−1.48	0.5	−0.03	S	Negative
PAO1 OXA-14	ESBL	D	1	−0.09	0.25	−0.18	S	Negative
PAO1 OXA-15	ESBL	D	1	−0.22	0.25	−0.12	S	Negative
PAO1 OXA-48	Carbapenemase	D	8	1.18	4	1.12	R	Positive

^*a*^ Description of the PAO1-derived isolate according to the β-lactamase produced.

^*b*^ β-lactam resistance phenotype.

^*c*^ MIC (mg/L) of imipenem (IMI) and imipenem/relebactam (IMR).

^*d*^ Ratio of hydrolysis (RH) of imipenem (IMI) and imipenem/relebactam (IMR) obtained by MALDI-TOF MS.

^*e*^ Interpretation of susceptibility/resistance of imipenem/relebactam (IMR) according to EUCAST guidelines.

^*f*^ Interpretation of the hydrolysis of imipenem/relebactam (IMR) by MALDI-TOF MS.

**TABLE 4:** Description of the 121 WGS-characterized clinical *P. aeruginosa* isolates

CODE^*a*^	β-lactam resistance phenotype	β-lactam resistome		ST^*c*^	IMI (mg/L)^*d*^	RH IMI^*e*^	IMR (mg/L)^*d*^	RH IMR^*e*^	IMR_EUCAST (R > 2 mg/L)^*f*^	IMR_MALDI^*g*^	Overall agreement^*h*^
		Acquired β-lactamases^*i*^	β-lactam resistance mutations^*b*^								
PAE-1	Mutational	-	OprD Q158fs; ParS L79P; PBP4 T428P	ST155	16	−0.35	1	−0.11	S	Negative	Yes
PAE-2	Mutational	-	OprD F84fs; ParS V216A; ParR K106fs; MexZ Y161ins; AmpD A96T; AmpDh2 V40I	ST308	16	−0.25	2	−0.05	S	Negative	Yes
PAE-3	Mutational	-	MexR R78*; OprD M358fs	ST274	16	−0.41	2	0.02	S	Negative	Yes
PAE-4	Mutational	-	ParR E214K; NalD L169P; AmpR D135N	ST2956	16	−0.38	1	−0.21	S	Negative	Yes
PAE-5	Mutational	-	OprD Q142*; MexZ G195D; AmpR G154R	ST175	16	−0.48	2	−0.43	S	Negative	Yes
PAE-6	Mutational	-	OprD Q142*; MexZ G195D; AmpR G154R	ST175	8	−0.41	1	−0.45	S	Negative	Yes
PAE-7	Mutational	-	MexR E27fs; OprM D482fs; OprD P146fs; ParE A41G; AmpR D135G; PBP3 R551L	ST390	16	−0.38	1	−0.47	S	Negative	Yes
PAE-8	Mutational	-	OprD Q67fs; MexZ G195D; AmpD T139M	ST175	16	−0.33	2	−0.37	S	Negative	Yes
PAE-9	Mutational	-	OprD A397fs; MexZ G195D	ST175	16	−0.41	2	−0.01	S	Negative	Yes
PAE-10	Mutational	-	MexR R63H; OprD G104fs; NalD L72P	ST274	16	−0.45	2	0.18	S	Negative	Yes
PAE-11	Mutational	-	MexB L936fs; OprD Q340*; ParS A149T; MexZ S202fs; AmpD Q51*	ST235	16	−0.72	2	−0.14	S	Negative	Yes
PAE-12	Mutational	-	OprD Q166*; MexZ G195D; AmpD Q55P	ST175	16	−0.09	2	−0.07	S	Negative	Yes
PAE-13	Mutational	-	OprD N112fs; ParS R7H	ST1754	32	0.00	2	−0.08	S	Negative	Yes
PAE-14	Mutational	-	OprD R102fs	ST1068	32	−0.06	2	−0.10	S	Negative	Yes
PAE-15	Mutational	-	OprD L92fs; AmpR D135N	ST649	32	−0.11	2	−0.14	S	Negative	Yes
PAE-16	Mutational	-	OprD K550*; MexZ I108S, A222G; AmpD P162L	ST261	8	−0.12	1	−0.15	S	Negative	Yes
PAE-17	Mutational	-	OprD L220fs; MexZ G195D; NalD A55fs; AmpD G156D	ST175	16	−0.20	2	−0.02	S	Negative	Yes
PAE-18	Mutational	-	NalC T50P; Mpl ΔT118	ST3153	1	0.08	0.25	0.01	S	Negative	Yes
PAE-19	Mutational	-	OprD G35fs; MexZ G195D; AmpD V10G	ST175	16	−0.09	2	−0.09	S	Negative	Yes
PAE-20	Mutational	-	MexB S1041E, V1042A; OprD L70fs; ParS R356L; MexZ D197fs; NalD G78fs; AmpC E247K; AmpD H36R; MexD S685G, S915A; ArmZ P244L	ST560	2	−1.03	0.25	−0.19	S	Negative	Yes
PAE-21	Mutational	-	MexR P37fs; OprD D61fs; AmpC E247K; PBP3 R504C; MexD A866V	ST3611	2	−0.98	1	−0.17	S	Negative	Yes
PAE-22	Mutational	-	AmpC ΔG229-E247; NfxB E55K, ∆L88-H90	ST175	0.5	−0.84	0.5	−0.15	S	Negative	Yes
PAE-23	Mutational	-	MexB T463A; ParR V55I, I80L; MexY R109H, G511D; MexZ H51R, L138R; NalD T11fs; AmpR D135N; AmpC F147L, G248S, D272N; AmpD G46S; MexD A496V	ST260	0.5	−0.48	<0,25	−0.27	S	Negative	Yes
PAE-24	Mutational	-	MexR N79S; MexB S583T, A707S, I963V, S1041E, V1042A; OprM Q43*; OprD Y225fs; ParS E90K, A115E; ParR P131L; MexY S48N, V174F, T472A, D502G, Q843R, I849V, A865D, T1034A; MexX A15T, A48V, A89V, D384S; MexZ E21D, L138R, E146A, F192L, L196I; PBP4 R475Q; NalD E21D; NalC A4V, A78T, M151V; PBP5 N86S, S355T; PBP2 A532V, L534P; Mpl Q347R, A404P; AmpR V291fs; AmpC P180L, Q204R, V239G, K396A; PBP3 L240V, I524T; AmpD R11H, A85S, S175P; OprJ A34V, D35G, A47V, V99I, D255A, I452V, E467Q, ∆L471, R478G; MexD G147D, A642D, H651R, A653V, T659S, S738N, S915A; MexC E152Q, V282I, N299S, G334A, V337A, E350K, NfxB E75G, S182T; ArmZ Q264L, V266A, V362I; AmpDh2 A8V, Q110K	ST175	16	−0.61	<0,25	−0.37	S	Negative	Yes
PAE-25	Mutational	-	AmpC P7S, A227T; AmpD V10G; NfxB ∆L88-H90	ST313	8	−0.71	1	−0.09	S	Negative	Yes
PAE-26	Mutational	-	MexA E249K; MexZ G195E; Mpl M38fs; AmpR Y92C; AmpC G242R; PBP3 P215L; ArmZ V266M	ST175	16	−0.02	<0,25	−0.13	S	Negative	Yes
PAE-27	Mutational	-	OprD Q158fs; ParS L79P; PBP4 T428P; Mpl V324E; AmpC D245N	ST155	16	−0.08	2	0.03	S	Negative	Yes
PAE-28	Mutational	-	MexR A108P; MexA I261V; OprD K268*; MexY D628H; MexX L21M; PBP4 G263D; AmpD R11fs	ST802	16	0.07	2	0.02	S	Negative	Yes
PAE-29	Mutational	-	MexR T130fs; OprD Y283fs; AmpD F146fs	ST4519	16	−0.17	2	−0.11	S	Negative	Yes
PAE-30	Mutational	-	MexR L43fs; OprD Q400fs; ParS R321H; PBP4 L145Q; Mpl P132L; AmpC E247K; MexD K500Q	ST775	8	0.08	2	−0.02	S	Negative	Yes
PAE-31	Mutational	-	MexB S1041E, V1042A; ParS P156L; MexX I50V; MexZ K27fs; Mpl F263L; AmpC ∆P243-G250; MexD S915A; ArmZ A262S	ST4518	64	−0.29	2	−0.15	S	Negative	Yes
PAE-32	Mutational	-	MexR L43fs; OprD Q400fs; ParS R321H; PBP4 L145Q; Mpl P132L; AmpC F147L, E247K; MexD K500Q	ST775	2	−0.08	2	−0.01	S	Negative	Yes
PAE-33	Mutational	-	MexR E27fs; MexB S1041E, V1042A; OprD N112fs; ParS R356L; PBP4 I356A; AmpC S254ins; MexD S915A; ArmZ P244L	ST560	1	−0.31	1	−0.21	S	Negative	Yes
PAE-34	Mutational	-	MexB S1041E, V1042A; MexZ ∆Q103; AmpC A227T, E247K	ST235	32	−0.13	2	−0.12	S	Negative	Yes
PAE-35	Mutational	-	OprD P116fs	ST275	32	0.04	2	−0.03	S	Negative	Yes
PAE-36	Mutational	-	MexB S1041E, V1042A; MexZ ∆Q103; AmpC T96I	ST235	4	0.06	2	−0.13	S	Negative	Yes
PAE-37	Mutational	-	MexB S1041E, V1042A; MexZ ∆Q103; AmpC E247K	ST235	8	−0.01	2	−0.18	S	Negative	Yes
PAE-38	Mutational	-	MexR R63C; MexB S1041E, V1042A; MexZ ∆Q103; AmpC E247K	ST235	8	−0.05	2	−0.18	S	Negative	Yes
PAE-39	Mutational	-	MexR I104fs; AmpDh3 ∆E185-R189; OprD W6*; AmpD R164H	ST244	16	0.19	2	−0.02	S	Negative	Yes
PAE-40	Mutational	-	MexA G355S; ParS S277N; MexZ A47V; PBP4 F324I; Mpl S131N, A428T; AmpD A29V, W97C; MexD T286M	ST2223	32	−0.01	2	−0.14	S	Negative	Yes
PAE-41	Mutational	-	OprD Y350*; AmpC G229S; AmpD W174*; MexD G290D	ST381	8	−0.04	2	−0.27	S	Negative	Yes
PAE-42	Mutational	-	MexB D783A; ParR V55I; MexX LG22C; MexZ S165*; NalC Q134fs; AmpD P147fs; OprJ V472A	ST439	16	−0.28	2	0.08	S	Negative	Yes
PAE-43	Mutational	-	OprD W277*; MexZ E120*; NalC R7fs	ST274	32	0.06	2	0.18	S	Negative	Yes
PAE-44	Mutational	-	OprD L70fs; MexZ G195E; AmpR D135N; AmpC E247K; MexC S382*; ArmZ V266M	ST175	16	−0.09	2	−0.14	S	Negative	Yes
PAE-45	Mutational	-	MexB S1041E, V1042A; OprD G124fs; ParS R356L; MexZ D197fs; NalD G78fs; AmpD G169D; MexD S915A; ArmZ P244L	ST560	16	−0.13	2	−0.40	S	Negative	Yes
PAE-46	Mutational	-	MexZ G137D; PBP4 T428P; NalD E141G; Mpl P274T, A450T; AmpD D28fs; OprJ A232T	ST938	32	−0.13	2	0.02	S	Negative	Yes
PAE-47	Mutational	-	OprD Q400fs; NalD L33P; AmpR D135N	ST274	32	−0.06	2	−0.23	S	Negative	Yes
PAE-48	Mutational	-	OprD Q400fs; NalD L33P; AmpR D135N	ST274	32	−0.20	2	−0.40	S	Negative	Yes
PAE-49	Mutational	-	OprM S169fs; ParS D24E; OprJ G334S; MexC Q381*; NfxB L14R	ST3225	16	−0.24	2	−0.31	S	Negative	Yes
PAE-50	Mutational	-	MexB S1041E, V1042A; OprD W417*; MexZ M34fs; NalD L100fs; Mpl FGG438ins	ST235	16	−0.10	2	−0.20	S	Negative	Yes
PAE-51	Mutational	-	MexR L123fs; AmpDh3 L132Q; E168D; OprD V127fs; ParS S277N; AmpD F3C; MexC V370A	ST1028	16	−0.06	2	−0.18	S	Negative	Yes
PAE-52	Mutational	-	OprD F36fs; MexZ L199Q; AmpD D135N	ST446	16	0.07	1	0.02	S	Negative	Yes
PAE-53	Mutational	-	MexB S1041E, V1042A; OprD W417*; MexZ M34fs; NalD L100fs; PBP3 R504C	ST235	16	−0.18	2	0.19	S	Negative	Yes
PAE-54	Mutational	-	OprD T181fs; ParS D68N; MexX V335M; AmpD W9fs	ST460	32	0.04	2	0.17	S	Negative	Yes
PAE-55	Mutational	-	AmpC P243S	ST170	4	−0.03	1	0.04	S	Negative	Yes
PAE-56	Mutational	-	OprD W65*; MexZ L199Q; NalD F51V; PBP2 P597Q; PBP3 F533L	ST446	16	−0.06	2	0.07	S	Negative	Yes
PAE-57	Mutational	-	OprD Q400fs; MexZ Q134fs; NalD L33P; AmpR D135N, S271L	NA	8	0.09	2	0.01	S	Negative	Yes
PAE-58	Mutational	-	OprD W65*; MexZ L199Q; NalD F51V; PBP2 P597Q; PBP3 F533L	ST235	16	−0.47	2	−0.11	S	Negative	Yes
PAE-59	Mutational	-	MexB S1041E, V1042A; OprD G124fs; ParS R356L; MexZ D197fs; NalD G78fs; PBP3 R504C; AmpD G169D; MexD S915A; ArmZ P244L	NA	16	−0.18	2	−0.19	S	Negative	Yes
PAE-60	Mutational	-	MexB S1041E, V1042A; OprD G124fs; ParS R356L; MexZ D197fs; NalD G78fs; PBP3 R504C, AmpD G169D; MexD S915A; ArmZ P244L	ST560	16	−0.39	2	−0.01	S	Negative	Yes
PAE-61	Mutational	-	MexB S1041E, V1042A; OprD D118fs; ParS R356L; MexX Y255*; PBP3 F533L; AmpD S102F; MexD S915A; ArmZ P244L	NA	8	−0.17	1	−0.12	S	Negative	Yes
PAE-62	Mutational	-	OprD S206fs; ParS Q349K; MexX Q343L; NalD Q35*; Mpl M38fs; OprJ A153T	ST2167	16	−0.48	2	−0.12	S	Negative	Yes
PAE-63	ESBL	PER-1, OXA-2	OprD A10fs; ArmZ Ter14Qext	ST235	8	−0.64	1	−0.06	S	Negative	Yes
PAE-64	ESBL	PER-1, OXA-2	OprD A10fs; ArmZ Ter14Qext	ST235	16	−0.71	2	−0.04	S	Negative	Yes
PAE-65	ESBL	OXA-10	OprD W417*; MexZ ∆V102-V105, L138R, N186S; PBP2 A481T; MexD S685G, S915A	ST253	8	−0.11	1	−0.18	S	Negative	Yes
PAE-66	ESBL	OXA-14	OprD W417*; MexZ ∆V102-V105, L138R, N186S; PBP2 A481T; MexD S685G, S915A	ST253	8	−0.37	1	−0.15	S	Negative	Yes
PAE-67	ESBL	OXA-794	OprD W417*; MexZ ∆V102-V105, L138R, N186S; PBP2 A481T; MexD S685G, S915A	ST253	8	−0.09	0.5	−0.06	S	Negative	Yes
PAE-68	ESBL	OXA-795	OprD W417*; MexZ ∆V102-V105, L138R, N186S; PBP2 A481T; MexD S685G, S915A	ST253	8	−0.33	1	0.02	S	Negative	Yes
PAE-69	ESBL	OXA-824	OprD W417*; MexZ ∆V102-V105, L138R, N186S; PBP2 A481T; MexD S685G, S915A	ST253	8	−0.52	0.5	−0.15	S	Negative	Yes
PAE-70	ESBL	PER-1	MexB S1041E, V1042A; OprD Q340*; ParS A149T; MexZ S202fs; AmpD Q51*	ST235	16	0.06	2	−0.08	S	Negative	Yes
PAE-71	Mutational	OXA-46	OprD L155fs; ParS L137P, H398R; PBP3 R504C	ST111	16	0.14	8	−0.11	R	Negative	No
PAE-72	Mutational	-	MexR Q106fs; OprD Y214*	ST274	32	−0.38	8	−0.21	R	Negative	No
PAE-73	Mutational	-	MexR V16fs; AmpDh3 D137fs; OprD W415*; MexZ G195D; AmpD F113L	ST175	16	−0.74	4	−0.17	R	Negative	No
PAE-74	Mutational	-	OprD G212fs; NalD Q126fs	ST235	16	−0.06	4	−0.05	R	Negative	No
PAE-75	Mutational	-	MexR R63H; PBP3 R504C	ST4520	32	0.08	4	−0.08	R	Negative	No
PAE-76	Mutational	-	OprD L195fs; PBP4 L178R; NalC KA174ins; AmpD G148A; ArmZ A262S	ST2570	16	−0.06	4	0.00	R	Negative	No
PAE-77	Mutational	-	OprD Q295fs; MexY A596V; NalD ∆N175-F184; AmpD L32fs; MexD P9A, L1027V; NfxB V134L	ST1247	32	0.11	4	0.00	R	Negative	No
PAE-78	Mutational	-	MexR I68L; OprD P186fs	ST564	16	−0.14	4	0.08	R	Negative	No
PAE-79	Mutational	-	MexR N79S; MexB S583T, A707S, I963V, S1041E, V1042A; OprM Q265E; OprD G178fs; ParS E90K, A115E; MexY S48N, T472A, D502G, R519H, Q843R, I849V, A865D; MexX A15T, A48V, E142K, D384G; MexZ E146A, F192L; PBP4 R475Q; NalD E21D; NalC H150fs; PBP5 N86S, S355T; PBP2 A272V, A532V, L534P; Mpl I266S, Q347R, A404P; AmpC Q204R, K396A; PBP3 L240V, A572V; AmpD R11H, A85S, S175P; OprJ A41T, I452V, E467Q, ∆L471; MexD G147D, A642D, H651R, T659S, S738N; MexC E152Q, V282I, N299S, G334A, V337A, E350K; NfxB E75G; ArmZ Q264L, V266A, V362I; AmpDh2 A8V, Q110K	NA	32	−0.15	8	0.11	R	Negative	No
PAE-80	Mutational	-	MexR Q106*; OprD W415*; MexY N1036S	ST274	32	−0.14	8	0.00	R	Negative	No
PAE-81	Mutational	-	MexR L102fs; OprD Y350*; ParS A345T; ParR ∆E214; AmpC E247G	ST2685	8	−0.06	4	0.05	R	Negative	No
PAE-82	ESBL	GES-1	OprD K2fs	ST235	16	−0.11	4	−0.08	R	Negative	No
PAE-83	ESBL	GES-1	MexZ E98fs	ST235	16	0.01	4	−0.21	R	Negative	No
PAE-84	ESBL	GES-7	MexR T130P; MexB S1041E, V1042A; ParS D149N; MexZ E98fs	ST235	8	−0.02	4	−0.05	R	Negative	No
PAE-85	ESBL	GES-1	MexB S1041E, V1042A; ParS D249N; MexZ E98fs	ST235	16	−0.31	4	−0.10	R	Negative	No
PAE-86	ESBL	GES-1	MexB S1041E, V1042A; ParS D249N; MexZ E98fs	ST235	16	−0.09	4	−0.11	R	Negative	No
PAE-87	Carbapenemase	IMP-8	-	ST155	64	0.53	64	0.61	R	Positive	Yes
PAE-88	Carbapenemase	VIM-20, OXA-2	OprD Q19fs; MexZ G195D; Mpl M38fs	ST175	64	1.04	64	0.55	R	Positive	Yes
PAE-89	Carbapenemase	IMP-8	IMP-8; OprD W339*; PBP4 S390R	ST155	>64	1.06	>64	0.95	R	Positive	Yes
PAE-90	Carbapenemase	VIM-2	ParS H194Q; MexZ L129fs; AmpD Q51*	ST235	>64	1.80	>64	2.98	R	Positive	Yes
PAE-91	Carbapenemase	VIM-2	ParS H398R, V152A; NalD N129K; NalC ∆C161-E165	ST973	>64	3.21	>64	4.47	R	Positive	Yes
PAE-92	Carbapenemase	VIM-2	OprD F256fs; ParS V152A; MexZ L93P; NalD V151fs; ArmZ S238ins	ST973	>64	2.97	>64	3.92	R	Positive	Yes
PAE-93	Carbapenemase	VIM-20, OXA-2	MexZ G195D	ST175	>64	1.56	>64	1.54	R	Positive	Yes
PAE-94	Carbapenemase	VIM-1	OprD W77*; MexZ V105G	ST253	>64	0.88	>64	0.76	R	Positive	Yes
PAE-95	Carbapenemase	IMP-1	MexA K86E; NalD V133fs; AmpD A134P; ArmZ A262S	ST664	8	1.65	8	1.30	R	Positive	Yes
PAE-96	Carbapenemase	VIM-2	ParS D380N; ParR I80L; ParR V55I; AmpD G46S	ST2136	16	3.03	16	1.86	R	Positive	Yes
PAE-97	Carbapenemase	VIM-20, OXA-210	OprD Q19fs; MexZ G195D; AmpD V10G	ST175	>64	1.21	>64	0.61	R	Positive	Yes
PAE-98	Carbapenemase	VIM-20, OXA-210	OprD Q19fs; MexZ G195D; AmpD V10G	ST175	>64	2.11	>64	2.47	R	Positive	Yes
PAE-99	Carbapenemase	VIM-20, OXA-2	MexZ G195E; AmpD D183T	ST175	>64	1.29	>64	2.47	R	Positive	Yes
PAE-100	Carbapenemase	VIM-20, OXA-2	OprD Q19fs; MexZ G195D	ST175	>64	0.70	>64	1.59	R	Positive	Yes
PAE-101	Carbapenemase	GES-5	OprD W277*; MexZ G195D	ST175	64	0.63	32	0.03	R	Negative	No
PAE-102	Carbapenemase	VIM-2	MexZ G195D	ST175	>64	1.04	>64	1.50	R	Positive	Yes
PAE-103	Carbapenemase	GES-5, GES-1, OXA-2	-	ST235	64	1.01	64	0.08	R	Negative	No
PAE-104	Carbapenemase	GES-5	MexZ E98fs; AmpD D112Y	ST235	32	0.08	16	0.16	R	Negative	No
PAE-105	Carbapenemase	VIM-2	ParS L137P, S228P; AmpD Q88L	ST111	>64	1.15	>64	1.25	R	Positive	Yes
PAE-106	Carbapenemase	IMP-33	-	ST111	>64	0.78	>64	0.82	R	Positive	Yes
PAE-107	Carbapenemase	VIM-2, CARB-2	MexR R23fs; OprD Q67fs; PBP5 I207fs	ST111	>64	1.34	>64	2.02	R	Positive	Yes
PAE-108	Carbapenemase	VIM-20, OXA-2	MexB L28fs; OprD Q19fs; MexZ G195E; AmpD T139K; ArmZ V266M	ST175	>32	0.66	64	1.38	R	Positive	Yes
PAE-109	Carbapenemase	VIM-20, OXA-681	OprD Q19fs; MexZ G195E; Mpl M38fs; ArmZ V266M	ST175	4	1.33	4	1.73	R	Positive	Yes
PAE-110	Carbapenemase	VIM-1	OprD W277*; MexZ V105G; PBP2 A481T; MexD S915A	ST253	>64	1.25	>64	0.97	R	Positive	Yes
PAE-111	Carbapenemase	VIM-1	OprD W277*; MexZ V105G; PBP2 A481T; MexD S915A	ST253	>64	0.70	>64	0.73	R	Positive	Yes
PAE-112	Carbapenemase	IMP-1	NalD V133fs; AmpD A134P; ArmZ A262S	ST664	8	1.00	8	0.76	R	Positive	Yes
PAE-113	Carbapenemase	IMP-16, OXA-129	MexZ ∆V102-V105, L138R, N186S; PBP2 A481T; MexD S915A	ST253	32	0.72	16	0.75	R	Positive	Yes
PAE-114	Carbapenemase	IMP-33	MexY G530S; MexD V864I	ST111	>64	0.75	>64	0.58	R	Positive	Yes
PAE-115	Carbapenemase	IMP-8	MexZ G50S	ST155	64	0.88	64	0.72	R	Positive	Yes
PAE-116	Carbapenemase	IMP-94, OXA-2	MexY V206L	ST303	8	1.09	8	1.35	R	Positive	Yes
PAE-117	Carbapenemase	GES-5	MexB S1041E, V1042A; ParS E219D	ST235	64	0.52	32	−0.01	R	Negative	No
PAE-118	Carbapenemase	GES-26, OXA-2	MexA K76Q; MexZ ∆E189-A194	ST309	64	0.34	32	0.00	R	Negative	No
PAE-119	Carbapenemase	GES-20, OXA-2	MexB S1041E, V1042A	ST235	32	0.57	32	0.14	R	Negative	No
PAE-120	Carbapenemase	VIM-1, GES-7	OprD Q400fs; MexZ V48A	ST155	>64	1.11	>64	1.48	R	Positive	Yes
PAE-121	Carbapenemase	GES-20, OXA-2	MexB S1041E, V1042A; ParS E219D	ST235	32	0.60	64	−0.10	R	Negative	No

^*a*^ Identification code.

^*b*^ fs indicates frameshift; * indicates stop codon; del indicates deletion; ins indicates insertion.

^*c*^ Sequence type (ST) obtained by WGS.

^*d*^ MIC (mg/L) of imipenem (IMI) and imipenem/relebactam (IMR).

^*e*^ Ratio of hydrolysis (RH) of imipenem (IMI) and imipenem/relebactam (IMR) obtained by MALDI-TOF MS.

^*f*^ Interpretation of susceptibility/resistance of imipenem/relebactam (IMR) according to EUCAST guidelines.

^*g*^ Interpretation of the hydrolysis of imipenem/relebactam (IMR) by MALDI-TOF MS.

^*h*^ Overall agreement between the phenotypic assay and the MALDI-TOF MS assay with respect to imipenem/ relebactam susceptibility prediction.

^*i*^ "-" are negative values.

#### Validation set

To further validate our algorithm in the clinical setting, we analyzed the performance of the proposed method using a collection of 250 clinical *P. aeruginosa* strains prospectively and randomly collected from patients admitted to the aforementioned hospitals (Table S2). Researchers were blinded to the MIC data for imipenem and imipenem/relebactam when conducting the MALDI-TOF spectra analysis. The presence of mutational mechanisms or carbapenemases in isolates showing imipenem or imipenem/relebactam resistance was determined using conventional phenotypic assays (cloxacillin inhibition tests and disk synergy assays with boronic acid, EDTA, and avibactam) (23, 24) and further confirmed by genotypic methods (PCR plus Sanger sequencing for detection of acquired β-lactamases), if phenotypic screening was positive (25).

### WGS and resistome analysis

Bacterial isolates had been previously sequenced or were sequenced *ad hoc* for this work using previously described methodologies. For each isolate, genomic DNA was extracted, and indexed paired-end libraries were generated using the Illumina DNA Prep library preparation kit (Illumina Inc, USA). WGS was performed on an Illumina MiSeq platform. The resulting reads were assembled using Unicycler (v.0.5.0) (26), and *de novo* assemblies were used to define the MLST (Center for Genomic Epidemiology, v2.0) (27). To identify horizontally acquired antimicrobial resistance genes, the online RGI (v.5.2.0) and CARD (v.3.2.8) tools were used, with default options (28). To characterize the mutational mechanisms involved in β-lactam resistance, the presence of mutations in a previously developed list of up to 48 chromosomal genes was analyzed, and common polymorphisms were filtered using the list of mutations previously developed by Cortés-Lara *et al*. (29). Mutations in the outer membrane porin OprD were manually reviewed using assembled genomes according to their similarity to the *oprD* sequence of strains PAO1, LESB58, UCBP-PA14, MTB-1, FRD1, or F23197, as previously described.

### Antimicrobial susceptibility testing

The MICs of imipenem and imipenem/relebactam for all isolates included in the study were determined as the modal MIC value obtained in triplicate experiments by reference broth microdilution assays in cation-adjusted Müeller–Hinton (MH) broth according to CLSI M100 guidelines. In all cases, relebactam was tested at a fixed concentration of 4 mg/L. EUCAST v 14.0 clinical breakpoints were used as the main reference and CLSI (M100 Ed34) Performance Standards for Antimicrobial Susceptibility Testing as a comparator (30, 31). According to EUCAST guidelines, the resistant breakpoint is defined as >4 mg/L for imipenem and >2 mg/L for imipenem/relebactam. The CLSI guidelines establish a breakpoint of ≤2 mg/L for susceptibility, =4 mg/L for intermediate susceptibility, and ≥8 mg/L for resistance, for both imipenem and imipenem/relebactam. Reference strains *E. coli* ATCC 25922 and *P. aeruginosa* ATCC 27853 were used as controls in all experiments. The acceptable ranges considered for imipenem were from 0.06 mg/L to 0.5 mg/L in *E. coli* ATCC 25922 and from 1 mg/L to 4 mg/L in *P. aeruginosa* ATCC 27853. The acceptable ranges considered for imipenem/relebactam were from 0.06 mg/L to 0.5 mg/L in *E. coli* ATCC 25922 and from 0.25 mg/L to 1 mg/L in *P. aeruginosa* ATCC 27853.

### MALDI-TOF MS assay

**Fig 1:**
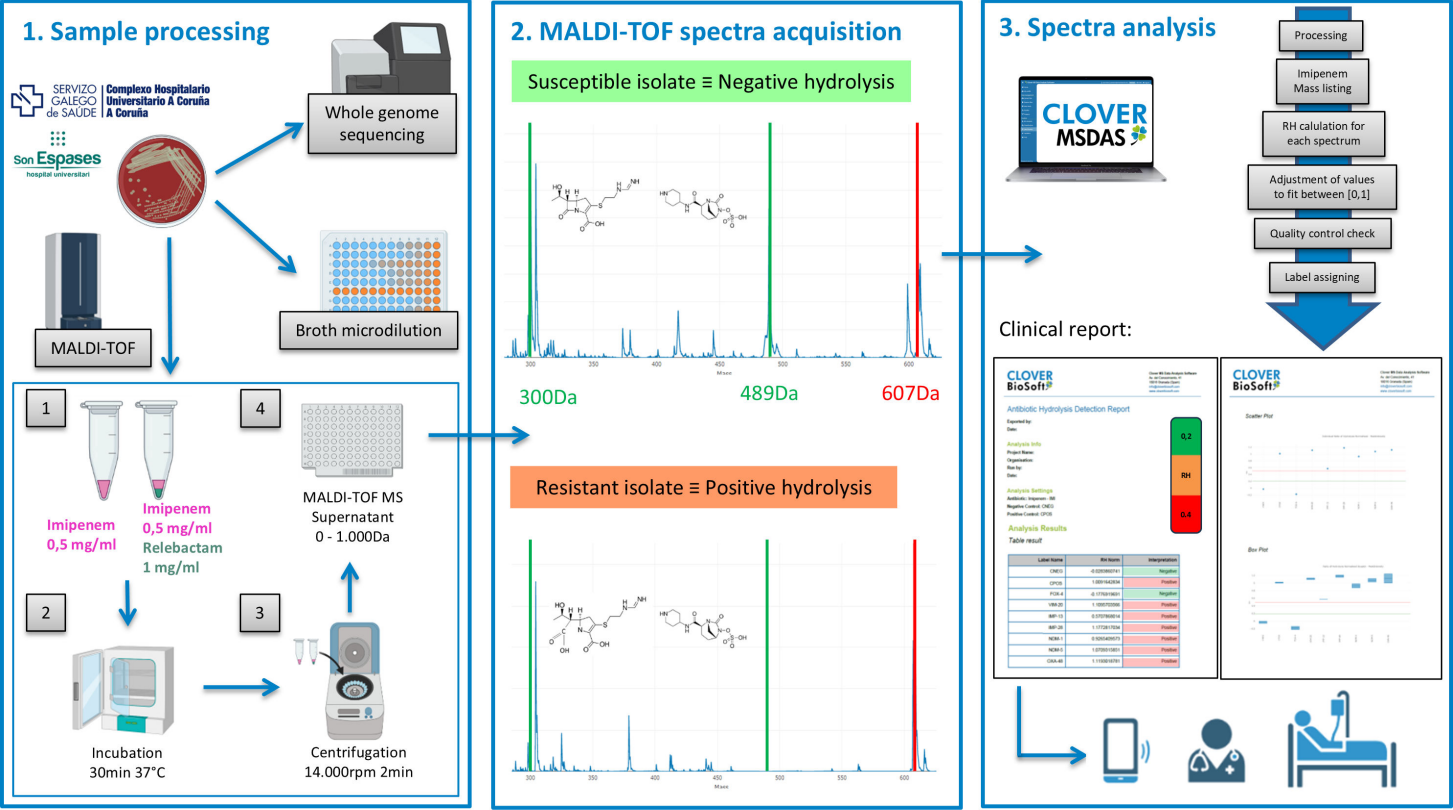
Workflow of MALDI-TOF samples is obtained from imipenem/relebactam susceptibility 1. Sample processing: samples are obtained from infected patients; pathogens are cultured and further genotypically and phenotypically characterized. The same isolates are analyzed by MALDI-TOF MS. 2. Spectra acquisition: The mass spectra profiles of imipenem and imipenem/relebactam are obtained by MALDI-TOF MS and uploaded within the Clover MSDAS platform to produce an automated susceptibility result. 3. Spectra analysis: The mass spectra profiles undergo preprocessing, imipenem mass listing, RH calculation, normalization, quality control checks, and label assignment, and a clinical report is finally provided within minutes with a table of results and a scatter plot and box plot for further imaging. Results are ready for clinical validation.

The MALDI-TOF MS and data processing workflow is summarized in Fig. 1. Briefly, bacteria filling a 1 µL inoculation loop were suspended in 50 µL of solution (10 mM NH_4_CO_3_, 10 µg/ml ZnCl_2_, 0.001% SDS; pH 8) containing 0.5 mg/mL of imipenem (Sigma Aldrich, Germany) and 0.5 mg/mL of imipenem with different concentrations of relebactam (4, 1, 0.5, and 0.25 mg/L) and incubated for 30 minutes at 37°C with slow agitation (300 rpm) until optimization of the protocol (14). The samples were then centrifuged at 16,800 x *g* for 2 minutes, and 1 µL of the supernatant was applied to a MALDI-TOF MS target plate. Each sample was spotted on the plate in duplicate. Once the spots had dried, 1 µL of the matrix [Matrix IVD HCCA-portioned (Bruker Daltonik) spiked with 1 ppm/ µL of reserpine (Sigma Aldrich, Germany)] was applied to each. All runs were performed in the presence of a positive and negative control treated in the same way as the samples. *P. aeruginosa* PAO1 was used as a negative control, and a previously constructed PAO1 strain expressing the *bla*_VIM-1_ gene in the pUCP24 plasmid was used as a positive control. Appropriate calibration was conducted before each run. The mass spectra were obtained using a MALDI Biotyper Smart (Bruker Daltonik) system, with Flex Control 3.4 software. The operational mass range was between 100 and 1,000 m/z in the linear positive mode. The mass peaks were acquired in 40 shot steps to produce 240 satisfactory shots, and the resolution of the mass peaks selected was higher than 300. The movement of the laser in the spot followed a large spiral. The overall procedure from the preparation of reagents until the results are obtained is done within 1 hour.

### MALDI-TOF MS data processing and interpretation

The principle of the MALDI-TOF MS assay is the inactivation of imipenem by carbapenemase-carrying bacteria due to hydrolysis of the β-lactam ring. The hydrolysis reaction modifies the structure of imipenem, causing a mass shift that can be detected by MALDI-TOF. In addition, parallel analysis of the blockage of this reaction can be performed when β-lactamase inhibitors are added (as in this case for relebactam). The Clover MS Data Analysis Software (Clover MSDAS) platform (Clover Biosoft, Spain) was used to evaluate the spectra. The software automatically calculates the RH value (which indicates the rate of hydrolysis) for imipenem in the presence or absence of relebactam. As each sample was spotted in duplicate, the final RH result is averaged. For categorical classification, we used the cut-off values recently developed by our group in Enterobacterales (14) and optimized in-house for *P. aeruginosa*. RH values below 0.2 represent no proven imipenem hydrolysis. RH values close to or above 0.5 indicate proven imipenem hydrolysis, and values between 0.2 and 0.5 represent an ambiguous hydrolysis pattern, which requires further testing or confirmation by other techniques. Thus, three potential scenarios can be observed when monitoring the degradation of both imipenem and imipenem/relebactam: i) absence of hydrolysis in both imipenem and imipenem/relebactam, indicating carbapenem susceptibility or carbapenem resistance through mutational events, and in both cases imipenem/relebactam susceptibility; ii) detectable hydrolysis in the imipenem assay that is blocked by the addition of relebactam (e.g., KPC production); and iii) imipenem hydrolysis not susceptible to relebactam inhibition: production of metallo-β-lactamases.

## RESULTS

### Optimization and performance of the imipenem/relebactam hydrolysis assay

**Fig 2:**
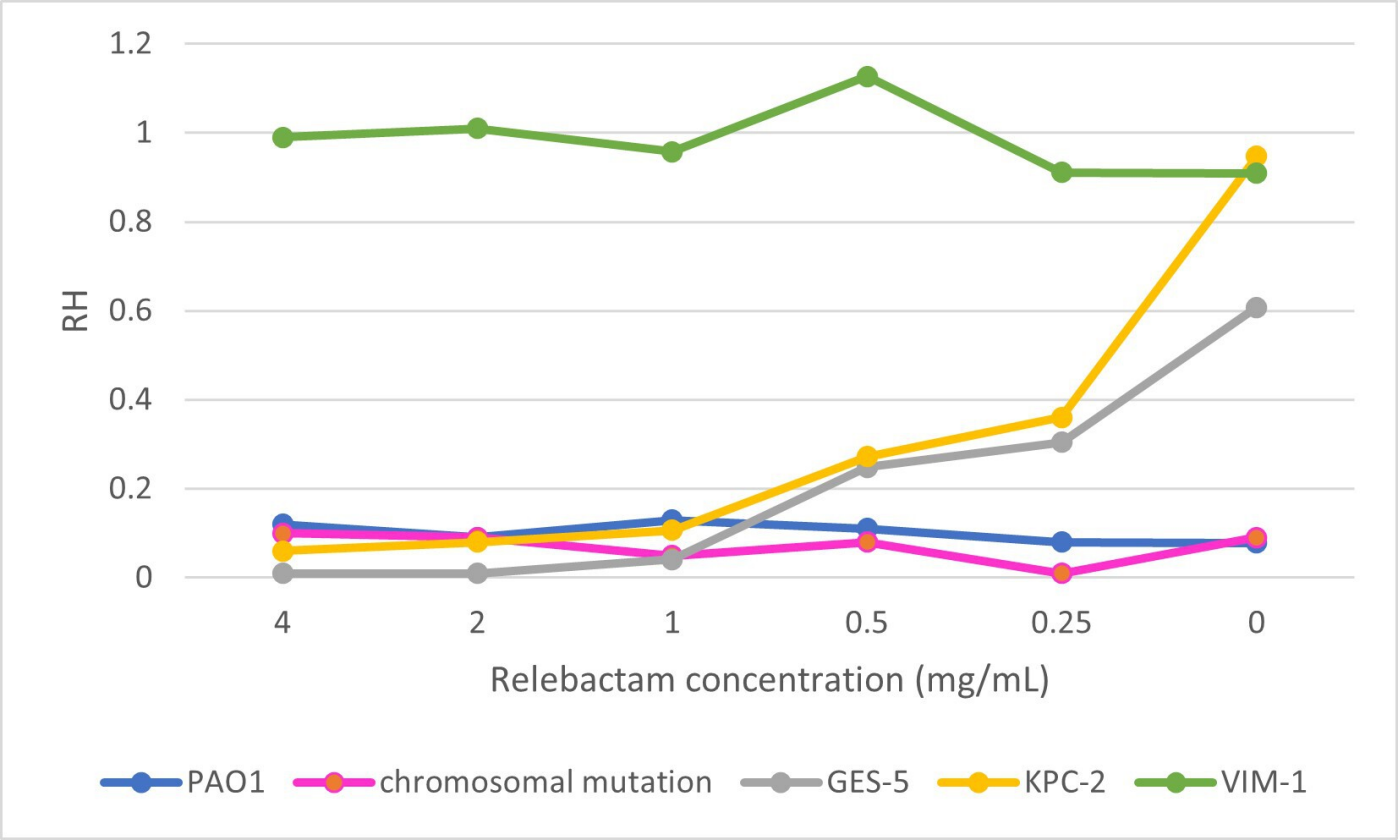
Variation of the MALDI-TOF MS RH for imipenem with different concentrations of relebactam.

The first step was to calculate the concentration of relebactam needed to block target enzymes. For this purpose, the imipenem hydrolysis assay was performed in combination with various concentrations of relebactam (4, 1, 0.5, and 0.25 mg/L). The assay was performed in triplicate using the following characterized control strains covering the different imipenem and imipenem/relebactam antimicrobial susceptibility and MALDI-TOF spectra profiles: the *P. aeruginosa* PAO1 reference strain, the PAO1 double *dacB* and *oprD* knockout mutant, and three PAO1 transformants expressing the *bla*_KPC-2_, *bla,*_GES-5_ and *bla*_VIM-1_ β-lactamases in the pUCP24 plasmid. The concentration of relebactam needed to decrease the RH to below 0.2 was 1 mg/L, decreasing in a proportional manner as higher concentrations of the inhibitor were added (Fig. 2). Thus, 1 mg/L was established as the optimal relebactam concentration for validation purposes. The second step was to identify the optimal hydrolysis cut-off values for detection of imipenem hydrolysis from those previously established for Enterobacterales by our group (14). We validated this method internally with *P. aeruginosa* as the target pathogen using the PAO1 reference strain as a negative control and a PAO1 transformant expressing the *bla*_VIM-1_ carbapenemase gene in the pUCP24 plasmid as a positive control. A receiver operating characteristic (ROC) curve was calculated by using the intensity of the imipenem mass peaks with a 95% CI. The cut-off values for the in-house developed assay were determined using the ROC curve and the Youden index (0.940), yielding a final value of RH ≥0.5 for positivity and <0.2 for negativity. RH values are normalized according to the positive and negative control.

### Preclinical evaluation

#### Evaluation of the algorithm with isogenic laboratory strains showing the most important mutational and transferable β-lactam resistance mechanisms found in *P. aeruginosa*

Our method was first evaluated with our previously constructed isogenic laboratory collection of PAO1 derivatives (Tables 2 and 3). Analysis of the laboratory isolates exhibiting mutation-driven resistance mechanisms (imipenem/relebactam MICs ranging from 0.125 to 1 mg/L) revealed the absence of detectable hydrolysis for both imipenem and imipenem/relebactam in all cases, successfully predicting non-carbapenemase production with 100% accordance (17/17). Additionally, imipenem/relebactam susceptibility was also predicted with 100% correlation (17/17).

Second, our algorithm also performed well with the isogenic transformants expressing the major β-lactamases found in *P. aeruginosa*, providing strong evidence for the potential application of the method to rapidly distinguish between carbapenemase-producing and non-producing *P. aeruginosa* isolates. In this context, transformants expressing β-lactamases associated with a cephalosporin resistance phenotype (such as OXA-2, OXA-10, VEB-1, PER-1, GES-1, KPC-31, and KPC-35) consistently showed the absence of hydrolysis with both imipenem and imipenem/relebactam, thus correctly predicting the absence of carbapenemases in 100% of cases (31/31). Moreover, analysis of the performance with carbapenemase-producing isolates also yielded promising results, 85% (12/14), resulting in protection against imipenem hydrolysis when relebactam was added against KPC producers, but not against those transformants carrying VIM, IMP, NDM, or OXA-48-type enzymes, the latter of which are resistant to relebactam inhibition. In the strains producing GES-5 and GES-20, hydrolysis of imipenem or the imipenem/relebactam combination was not observed, and thus MALDI-TOF MS was not able to recognize the presence of these carbapenemase enzymes. Imipenem/relebactam susceptibility was correctly predicted (100%, 31/31) for all the β-lactamase transformants evaluated, including GES carbapenemase producers (in these latter, the results were consistent with the susceptibility profile as both isolates showed susceptibility to imipenem and imipenem/relebactam).

#### Evaluation of the algorithm with WGS-characterized MDR/XDR clinical isolates of *P. aeruginosa*

The imipenem/relebactam MALDI-TOF MS-based assay was tested on a collection of 121 MDR/XDR *P. aeruginosa* strains. For these challenging strains, the method accurately detected carbapenemase production in 80% (28/35) of the isolates. Errors were observed in the seven GES-producing isolates (four GES-5 and three GES-20). Class B carbapenemases were correctly detected in all cases (28/28), confirming the reliability of this method for predicting metallo-β-lactamases. For GES-positive strains, hydrolysis of imipenem/relebactam was not detected in any case, while hydrolysis of imipenem was detected in five cases, suggesting possible inhibition by relebactam. However, the imipenem hydrolysis ratio for the GES-5 and GES-20 carbapenemases was very close to the cut-off, with an average RH of 0.53. Despite the low carbapenemase activity detected by MALDI-TOF, the imipenem MICs of these strains ranged from 16 to 64 mg/L. Furthermore, the method demonstrated an 81% (98/121) global agreement between the imipenem/relebactam susceptibility profile and the MALDI-TOF MS hydrolysis result (Table 4). Susceptible isolates, as determined by reference broth microdilution, showed 100% (70/70) agreement with the MALDI-TOF MS assay, whereas resistant isolates showed only 54.9% (28/51) agreement. The errors were primarily associated with GES-producing isolates (12 expressing GES-1, GES-5, GES-7, or GES-20) in combination with additional mutational resistance mechanisms, such as OprD inactivation, and with another 11 isolates expressing resistance (MICs ranging from 4 to 8 mg/L) due to different combined mutational mechanisms.

#### Validation of the algorithm with clinical isolates

Among the 250 prospectively collected clinical isolates, the method demonstrated 100% (9/9) accuracy in detecting the presence of carbapenemases (one IMP and eight VIM producers), particularly metallo-β-lactamases in a real-life scenario. Thus, the method displayed a 100% (241/241) accuracy for ruling out carbapenemases. In addition to these nine carbapenemase-producing strains, four other isolates showed imipenem/relebactam resistance (in all cases with an MIC of 4 mg/L) but lacked carbapenemases, resulting in a total of 13 prospectively collected imipenem-/relebactam-resistant strains. This brings an overall performance of the method for predicting imipenem/relebactam resistance in prospectively collected strains of 70% (9/13).

A detailed analysis of the agreement between the method’s prediction of the imipenem/relebactam susceptibility profile revealed that the errors in predicting imipenem/relebactam resistance in non-carbapenemase-producing isolates had a minor impact on the overall agreement (98%, 246/250). In these cases, although the algorithm failed to predict the susceptibility profile, the underlying mechanisms of resistance to β-lactams were correctly inferred (resistance due to mutational events). For the remaining 237 imipenem-/relebactam-susceptible isolates, no hydrolysis of the antibiotic combination was observed in any case (100%, 237/237).

To assess the impact of these errors, the isolates were grouped by susceptibility categories according to their MIC. In addition to using the primary comparator for susceptibility categorization (EUCAST guidelines), we also applied the CLSI guidelines (Table 5). Very major, major, and minor errors were defined. No major errors were observed according to either EUCAST or CLSI guidelines. However, while EUCAST guidelines indicated 31% (4/13) of very major errors, CLSI classified these as minor errors, with 2% (4/250) of isolates misclassified as susceptible by MALDI-TOF being categorized as intermediate in the CLSI classification.

**TABLE 5:** Incidence of *Pseudomonas aeruginosa* clinical isolates in qualitative imipenem/relebactam susceptibility categories according to broth microdilution testing and errors associated with the MALDI-TOF MS prediction algorithm

Interpretative criteria		MALDI-TOF MS susceptibility ^*a*^result		Error classification^*c*^
		Incidence of isolates (n)		
		S	R	
EUCAST	S^*b*^	237	0	Major error 0%
	R^*b*^	4	9	Very major error 31%
CLSI	S	237	0	Minor error 2%
	I	4	0	Major error 0%
	R	0	9	Very major error 0%

^*a*^ MALDI-TOF MS susceptibility is indicated by an imipenem/relebactam ratio of hydrolysis (RH) < 0.2 and resistance is indicated by an RH≥ 0.5.

^*b*^ R, resistant; S, susceptible; I, intermediate resistance.

^*c*^ “Very major error” (false susceptibility) indicates that the isolate is categorized as susceptible by MALDI-TOF MS and resistant by the reference method; “major error” (false resistance) indicates that the isolate is categorized as resistant by MALDI-TOF MS and as susceptible by the reference method; “minor error” indicates that the isolate is categorized as intermediate by one method and resistant or susceptible by the other.

## DISCUSSION

In this work, we took advantage of well-characterized (including by WGS) collections of laboratory isolates and clinical strains, and of the high activity and particular mode of action of imipenem/relebactam against *P. aeruginosa,* to develop a MALDI-TOF-based methodology that provides presumptive information about the underlying carbapenem resistance mechanisms. The proposed method could therefore represent a valuable tool for the rapid detection of carbapenemases and thus help in the establishment of targeted imipenem/relebactam treatments to combat *P. aeruginosa* infections in certain clinical scenarios. In the last decade, several MALDI-TOF-based methods aimed at monitoring the hydrolysis of different β-lactam substrates (e.g. cefotaxime and imipenem) for the early detection of ESBLs or carbapenemases have been developed (13). These methods have been evaluated in growing colonies and also in clinical samples such as urine or blood cultures to improve decision-making about treatment of severe infections (32). Moreover, the correlation of these methodologies with genomic data and antimicrobial susceptibility testing results has been explored in limited detail previously. This is attributed to the fact that the methods have been extensively applied to Enterobacterales, in which (unlike in *P. aeruginosa*) resistance is commonly caused by horizontally acquired enzymes, such as OXA-48, which produces positive carbapenem hydrolysis but commonly yields low carbapenem MICs (even below the susceptibility breakpoint) (33).

Regarding the performance of the proposed method, analysis of the data obtained from the training set of strains demonstrated promising results, as exemplified by the 95.8% (46/48) agreement in predicting carbapenemases with the *P. aeruginosa* PAO1 mutants (Table 2) and the PAO1 β-lactamase-producing transformants (Table 3). This was particularly evident for KPC β-lactamases, which were inhibited by relebactam, or the metallo-β-lactamases, which conferred observable hydrolysis with and without relebactam. These findings are encouraging as KPC is an emerging mechanism among *P. aeruginosa* strains worldwide, particularly in China (34), whereas metallo-β-lactamases are globally the most widely scattered transferable resistance determinants in *P. aeruginosa* (35). Intriguingly, the expression of both GES-5 and GES-20 carbapenemases (as confirmed by PCR and phenotypic assays with the resulting transformants) in PAO1 did not confer imipenem resistance (indicated by MICs), and hydrolysis was also undetectable by MALDI-TOF. Notably, this association between low carbapenem MICs and inconsistent hydrolysis has already been described for GES-type β-lactamases. Recent research with PAO1 isogenic transformants revealed that overexpression of GES-type β-lactamases in *P. aeruginosa* using a multicopy plasmid was not able to increase the MIC of imipenem further than 2 mg/L (36), which may be related to the low *K*_cat_/K_m_ values obtained with purified GES-5 β-lactamase in previous experiments (37). These observations are also consistent with the contradictory results obtained with GES-positive strains in commercially available methods aimed at detecting carbapenem hydrolysis in clinical microbiology laboratories, such as CarbaNP or β-Carba tests, in which these types of enzymes persistently yield negative hydrolysis results (38). Interestingly, the method also accurately inferred imipenem/relebactam susceptibility in these collections of isogenic strains.

In a second step, further analysis of the performance of the algorithm with the challenge set of 121 MDR/XDR WGS-characterized *P. aeruginosa* strains (Table 4) revealed 80% (28/35) accuracy for predicting carbapenemases. Again, inconsistent results were obtained for GES-producing strains. Fortunately, the global prevalence of these enzymes among carbapenem-resistant *P. aeruginosa* strains is low, but the findings suggest that the implementation of this methodology should be carefully monitored in clinical environments with a high prevalence of GES-producing *P. aeruginosa*. On the other hand, the method demonstrated 81% (98/121) accuracy for predicting the imipenem/relebactam susceptibility profile. This percentage of susceptibility was also influenced by GES-producing *P. aeruginosa* but also by a cluster of 11 isolates, which showed imipenem/relebactam resistance (MIC = 4–8 mg/L) due to a combination of mutational events, with in all cases amino acid substitutions affecting OprD (10/11), or the *mexAB-oprM* (10/11) or *ampC* (5/11) regulatory pathways. Primary infections by *P. aeruginosa* isolates showing mutational resistance to imipenem/relebactam are infrequent. However, we recently reported the development of imipenem/relebactam resistance in MDR/XDR *P. aeruginosa* infections receiving long treatment courses with broad-spectrum antipseudomonals, due to selection of multiple resistance mechanisms, such as OprD inactivation in combination with AmpC and MexAB-OprM overproduction (39). Thus, this method should also be used with caution for suspected imipenem/relebactam susceptibility/resistance in patients who have been receiving prolonged treatment with carbapenems or have a history of previous MDR/XDR *P. aeruginosa-*positive cultures.

Finally, analysis of the performance of our method in a real-world scenario with prospectively collected clinical strains (Validation Set, Table S2) revealed 100% accuracy (9/9) in predicting carbapenemases, specifically metallo-β-lactamases. The method also demonstrated 98% agreement with the proposed algorithm (Table 1), suggesting that MALDI-TOF-based applications like this could be further developed for predicting imipenem/relebactam susceptibility in the future. However, when specifically evaluating the errors of the methodology following EUCAST guidelines, the very major error rate increased to 31% (4/13). This high percentage of error would be explained by the fact that isolates with mutational imipenem/relebactam resistance and showing an MIC of 4 mg/L are assigned to the susceptible category (the method assumes that isolates that do not hydrolyze either imipenem or imipenem/relebactam are susceptible to imipenem/relebactam due to their high activity against strains with mutational mechanisms). Interestingly, although the error rate of the MALDI-TOF assay seems beyond the possibility of clinical application, if CLSI breakpoints were applied, very major errors would be considered minor errors (2%, 4/250) since isolates with a 4 mg/L MIC would be considered resistant by EUCAST and intermediate by CLSI. Therefore, clinical application of the MALDI-TOF MS assay could be supported if following CLSI guidelines.

The results of this methodology are provided in the Clover MS Data Analysis Software (Clover MSDAS) including a laboratory report with the following: i) the ID of the isolate; ii) the average imipenem and imipenem/relebactam RH value; iii) and the interpretation of the RH (positive/negative) in relation to the presence/absence of carbapenemases. The following footnote at the bottom of the inform is included: “A positive imipenem/relebactam RH value is related with clinical resistance to the antibiotic combination. The accuracy of this method for carbapenemase detection may be limited in epidemiological scenarios with a high prevalence of GES-producing and also in MDR/XDR *P. aeruginosa* isolates.”

The method is simple, rapid, and combines the universal advantage of phenotypic assays with the speed of molecular assays, allowing results within 1 hour. This is at least between 1 and 15 hours faster than the currently available imipenem/relebactam testing methods in clinical microbiology laboratories, such as disk diffusion, MIC strips, broth microdilution (40), or the recently developed Rapid IPR NP test method (41). Therefore, apart from limitations that may affect the reliability of the method, such as a high prevalence of GES-type carbapenemases or strains resistant to imipenem/relebactam through chromosomal mutations, this methodology provides an additional tool that may aid in combating *P. aeruginosa* infections with imipenem/relebactam.

## Data Availability

The WGS data of the strains analyzed have been deposited in the European Nucleotide Archive (PRJEB31047) under the following accession numbers: ERS3119946 to ERS3120130 and in the GenBank databases (PRJNA523008 and PRJNA1133624).

## SUPPLEMENTAL MATERIAL

Table S1 jcm.01105-24-s0001.xlsx Description of the 121 WGS-characterized clinical *P. aeruginosa*.

Table S2 jcm.01105-24-s0002.xlsx Description of the 250 prospectively collected clinical *P. aeruginosa*.

ASM does not own the copyrights to Supplemental Material that may be linked to, or accessed through, an article. The authors have granted ASM a non-exclusive, world-wide license to publish the Supplemental Material files. Please contact the corresponding author directly for reuse.
